# Reduced contrast sensitivity function and outer retina thickness in convalescent Vogt-Koyanagi-Harada disease

**DOI:** 10.1038/s41433-024-03418-1

**Published:** 2024-11-08

**Authors:** Yi-Sha Li, Xia Hu, Fang-Yue Zhou, Xingneng Guo, Xiaoling Yang, Ruru Liu, Dan Lin, Mali Dai, Ke Wu, Jiaqing Wu, Fang Hou, Luis Andres Lesmes, Zhong-Lin Lu, Yu-Qin Wang

**Affiliations:** 1 National Clinical Research Center for Ocular Diseases, Eye Hospital, Wenzhou Medical University, Wenzhou, China; 2 The First Affiliated Hospital of Ningbo University, Ningbo, Zhejiang China; 3 Zhongshan Ophthalmic Center, Sun Yat-Sun University, Guangzhou, Guangdong China; 4 The Fourth Affiliated Hospital of Zhejiang University School of Medicine, Jinhua, China; 5 State Key Laboratory of Ophthalmology, Optometry and Vision Science, Eye Hospital, Wenzhou Medical University, Wenzhou, China; 6 Adaptive Sensory Technology Inc, San Diego, CA USA; 7 Division of Arts and Sciences and NYU-ECNU Institute of Cognitive Neuroscience, NYU Shanghai, Shanghai, China; 8 Center for Neural Science and Department of Psychology, New York University, New York, NY USA

**Keywords:** Autoimmune diseases, Uveal diseases

## Abstract

**Background:**

To evaluate contrast sensitivity function (CSF) in convalescent Vogt-Koyanagi-Harada (VKH) disease and investigate the relationship between CSF and chorioretinal thickness in VKH patients with and without sunset glow fundus (SGF).

**Methods:**

This is a cross-sectional study. Seventy-six eyes of VKH patients and 56 eyes of normal controls were evaluated. Patients were divided into SGF and non-SGF groups. The best-corrected visual acuity (BCVA) of all the participants was ≤0.0 logMAR. Their CSF and macular chorioretinal thickness were measured with quantitative CSF (qCSF) and Optical Coherence Tomography (OCT) and compared using repeated measures analysis of variance at the group level. Relationships between CSF and macular chorioretinal thickness were evaluated using generalized estimating equations.

**Results:**

The CSF was significantly impaired in the SGF group compared to that in the control group (*p* = 0.001), especially at medium and high spatial frequencies. No significant CSF difference was found between the non-SGF group and control group, nor between the SGF and non-SGF groups. Compared to the controls, outer retinal thickness (ORT) in both VKH subgroups was significantly reduced (*P* < 0.001 or 0.005, respectively), although their outer nuclear layer thickness (ONLT) and choroidal thickness (CT) were not significantly different (both *P* = 1.000, *P* = 0.829 or 0.112, respectively). We found no significant correlation between CSF metrics and outer retinal thickness.

**Conclusions:**

Despite good recovery of visual acuity, reduced CSF and outer retina thickness were found in convalescent VKH patients. CSF may be an important and sensitive metric to evaluate functional vision in VKH disease.

## Background

Vogt-Koyanagi-Harada (VKH) disease is an autoimmune disorder that affects the eyes, auditory systems, meninges and skin [1, 2]. It is one of the most common uveitis entities in China, accounting for about 12.7% of uveitis patients [3]. The chronic recurrent inflammation caused by VKH can lead to serious visual impairments [4–7]. With adequate systemic immunosuppressive therapy, VKH disease can usually develop into the convalescent stage and exhibit a good prognosis [1]. For most patients, vision and retinal structure can be restored as inflammation subsides [8, 9]. However, even after adequate systemic immunosuppressive therapy, some patients in the convalescent stage develop sunset glow fundus (SGF) [10, 11], characterized by chorioretinal depigmentation [2]. Recent research found that subclinical inflammation persisted in convalescent VKH disease [12]. Clinically, patients with recovered visual acuity still complain about poor visual quality [13]. A more complete evaluation of visual function in convalescent VKH disease is necessary.

Visual acuity, a measure of spatial resolution of central vision at high contrast, is commonly used to evaluate functional vision in VKH patients [9]. However, it poorly reflects accurate visual functions in real life [14, 15]. Studies have found that although visual acuity returned to normal, recovery of other vision functions, including electrophysiology parameters, visual field and colour vision were delayed or incomplete in VKH patients [16, 17].

The contrast sensitivity function (CSF), a measure of visual sensitivity to patterns of a wide range of spatial frequencies at threshold contrast levels, provides a more comprehensive assessment of spatial vision and is correlated with daily visual functions in real life [18]. Many eye diseases exhibited contrast sensitivity function deficits in their early stage even when visual acuity was normal or nearly normal [19–21] or after recovery of visual acuity [22]. Whether CSF deficits remain in patients in the convalescent stage of VKH, especially those with SGF, is not clear [23].

In addition, it is well known that the integrity of chorioretinal structure was associated with the prognosis of VKH disease [24–26]. Previous studies reported that the subclinical inflammation in VKH disease destroyed the protective barrier formed by retinal pigment epithelium (RPE) [27], which may lead to damaged fundus microstructures, including discontinuous outer retina, RPE or focal atrophy and choroid thinning [28–31]. Lee et al. [4] reported that ocular complications, longer disease duration and uveitis recurrences led to higher degrees of SGF. Others suggested that chorioretinal thickness was critically correlated with ocular inflammation and macular function [28, 32]. Takahashi et al. [30] demonstrated that choroidal thickness (CT) in eyes with no or mild SGF was close to normal eyes, but eyes with severe SGF had thinner choroid than in controls. Zhou et al. [28] found that patients with inactive VKH but impaired outer retina and thickened RPE had poorer best-corrected visual acuity (BCVA) and worse retinal sensitivity. We hypothesized that convalescent VKH patients, even those with good visual acuity, may have CSF deficits, and their CSF may be correlated with SGF and chorioretinal thickness.

## Methods

### Subjects

The study protocol was approved by the institutional review board of the Affiliated Eye Hospital of Wenzhou Medical University and adhered to the tenets of the Declaration of Helsinki. Written informed consent including consent to publish photographs was obtained from every participant before the study. A total of 38 subjects (mean age 41.1 ± 9.7 years, 42% male) with diagnosis of convalescent VKH disease and 28 normal controls (mean age 38.5 ± 11.9 years, 32% male) participated in the study between June 2020 and September 2021 in the Affiliated Eye Hospital of Wenzhou Medical University. The normal controls were staff members and graduate students recruited from the eye hospital.

VKH disease was diagnosed according to revised diagnostic criteria established by the International Nomenclature Committee [33, 34]. All patients were treated with a 1–2 weeks initial course of oral corticosteroid (1–1.2 mg/kg/day) and tapered dosage following the resolution of inflammation. The treatment was usually discontinued after at least 12 months. In this study, we only recruited patients with good visual acuity, who had mild disease and were sensitive to systemic steroids therapy. Immunomodulatory agents were not used in this study according to the standard practice for acute VKH [35]. The convalescent stage was defined as: disease duration > 3 months, no acute anterior inflammation, and absence of posterior active inflammatory signs such as retinal detachment, cystoid macula oedema, or disc oedema. The duration was defined as the interval between symptom onset and the examination time. The inclusion criteria for the VKH patients were: (1) age from 18 to 65 years, (2) clinical diagnosis of convalescent VKH disease, (3) no obvious cataract or other ocular diseases, and (4) BCVA ≤ 0.0 logMAR. Exclusion criteria for the participants were: (1) any evidence of systemic disease or cognitive disorder that may impair vision, (2) −6.00 dioptres (D) >spherical equivalent refractive error (SE) > +3.00 D. The diagnosis of SGF was determined by two experienced uveitis clinicians (DL and MLD) based on fundus photographs (CR-2AF, Canon Inc., Japan). The colour fundus photographs were commonly used in the diagnosis of SGF [30, 36]. Any disagreement was adjudicated by a third specialist (YQW).

All participants received a complete ocular examination, including BCVA, slit-lamp examination, CSF (MCVM; Adaptive Sensory Technology, San Diego, California, USA), optical coherence tomography (OCT) (Heidelberg Engineering, Heidelberg, Germany) and axial length (Lenstar LS 900, Haag Streit, Koeniz, Switzerland). BCVA was measured with the standard logarithmic visual acuity chart. [37] All participants had a duochrome (red-green) test.

### Contrast sensitivity function

The CSF was measured with the Manifold Contrast Vision Meter (Adaptive Sensory Technology, San Diego, California, USA), which uses quantitative CSF, a Bayesian active learning algorithm, to measure the entire CSF curve [38]. Bandpass-filtered Sloan digits at varying sizes were used in the ten-alternative forced digit identification task [39]. Participants identified the digits monocularly with their best refractive correction in a dark room, at a viewing distance of 3 m. Both eyes were tested. No feedback was provided.

The MCVM reported CS at six spatial frequencies (1, 1.5, 3, 6, 12, 18 cycles per degree [cpd]), the area under log CSF (AULCSF), and CSF acuity. AULCSF was used as the summary statistic of the CSF [40, 41]. CSF acuity represented the high spatial frequency cutoff of the visual system [42].

### Optical coherence tomography

**Fig. 1:**
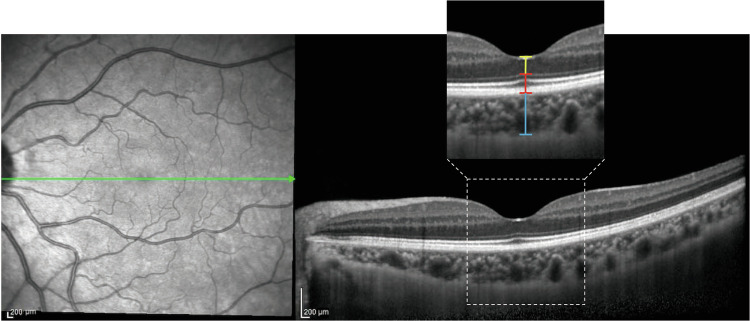
Schematic representation of macular structures. The right panels show the horizontal EDI-OCT image of the left eye of a 40-year-old healthy female. The yellow line indicates the subfoveal outer nuclear layer thickness, the red line indicates the subfoveal outer retinal thickness and the blue line represents the subfoveal choroidal thickness.

Chorioretinal images were taken by a trained ophthalmic photographer using the Heidelberg Spectralis OCT instrument (Heidelberg Engineering, Heidelberg, Germany). The enhanced depth imaging (EDI) mode, centred on the fovea and comprising 1536 A-scans per B-scan, was used, with the eye tracking system monitoring eye positions for clear images. All scans were performed by an experienced operator, who was blinded to the clinical data. Chorioretinal thickness was manually measured by two independent investigators (XNG, YSL) using the virtual ruler tool provided by the Heidelberg Spectralis. Manual measurements were commonly used for quantifying chorioretinal thickness [36, 43]. Subfoveal outer nuclear layer thickness (ONLT) was measured from the internal limiting membrane to the upper boundary of the outer limiting membrane. Subfoveal outer retinal thickness (ORT) was measured from the upper boundary of the outer limiting membrane to the inferior boundary of Bruch’s membrane. Subfoveal CT was measured from the inferior boundary of the Bruch’s membrane to the choroid–scleral interface (Fig. 1). The average thicknesses of two measurements from the OCT horizontal and vertical images were used for statistical analysis.

### Statistical analysis

Statistical analyses were performed using SPSS 19.0 (SPSS Science, Chicago, IL, USA). Continuous descriptive statistics were represented as mean ± standard deviation. Categorical variables were compared via the Chi-Squared test. Both eyes of the participants were included. Considering the possible correlation between the two eyes, group comparisons were performed using repeated measures analysis of variance (ANOVA) followed by post hoc analysis with Bonferroni correction. For the CSF, the effects of eye, spatial frequency (SF) and group were considered; For the other measures, the effects of eye and group were considered. The correlations between functional and structural metrics were evaluated using generalized estimating equations (GEE) with eye as a within-subject factor. *P*-value < 0.05 was considered statistically significant.

## Results

### Clinical characteristics

Seventy-six eyes of 38 VKH patients in the convalescent phase with BCVA ≤ 0.0 logMAR were examined in this study. Patients were divided into two subgroups, the SGF group with 34 eyes from 17 patients and the non-SGF group with 42 eyes from 21 patients. In addition, 56 eyes of 28 normal controls, matched for gender, age, visual acuity, and axial length, were examined. The baseline demographics and characteristics of the participants are described in Table 1. There was no significant difference between the control and VKG groups, nor among the control, non-SGF and SGF groups.

**Table 1:** Baseline demographics and characteristics of the eyes.

Parameters	Control	VKH	Non-SGF	SGF	*P*^a^ value	*P*^b^ value
Eyes	56	76	42	34		
Age (years)	38.5 ± 11.9	41.1 ± 9.7	42.5 ± 7.7	39.2 ± 11.7	0.351	0.324
Male/Female	9/19	16/22	9/12	7/10	0.410	0.708
Disease duration (months)	NA	21.6 ± 27.6	19.4 ± 19.2	24.4 ± 35.8	NA	0.977
BCVA (logMAR)	−0.03 ± 0.05	−0.04 ± 0.06	−0.04 ± 0.06	−0.03 ± 0.05	0.851	0.860
SE	−0.97 ± 1.67	−1.12 ± 1.72	−1.02 ± 1.93	−1.25 ± 1.44	0.472	0.148
Axial length (mm)	23.8 ± 1.0	23.5 ± 1.1	23.5 ± 1.3	23.5 ± 0.8	0.257	0.514

*SGF* sunset glow fundus, *BCVA* best-corrected visual acuity, *SE* spherical equivalent refractive error, *NA* not applicable.

^*a*^*P* = *P*-value between the control and VKH groups.

^*b*^*P* = *P*-value among the control, non-SGF and SGF groups.

### Contrast sensitivity dysfunction in VKH patients

**Fig. 2:**
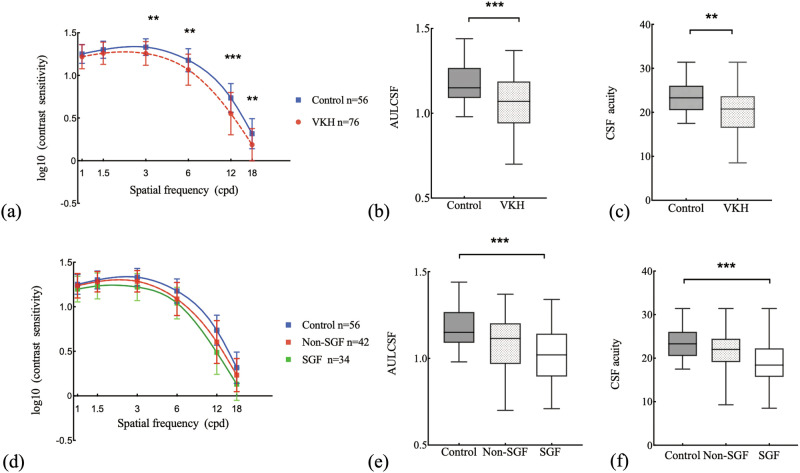
Contrast sensitivity functions of the convalescent VKH patients and controls. **a** Average contrast sensitivity functions of VKH patients and controls. VKH patients had lower CS, especially in medium and high spatial frequencies. **b**, **c** AULCSF and CSF acuity of the two groups. **d** Average contrast sensitivity functions of the SGF, non-SGF and control groups. **e**, **f** AULCSF and CSF acuity of the three groups. The central lines of the box plots represent medians. The boxes encompass the 25th–75th percentile, with whiskers representing the minimum and maximum. Error bars: standard deviation. *P < 0.05, **P < 0.01, and ***P < 0.001.

The average CSFs of the VKH patients and controls are plotted in Fig. 2a. Repeated measures ANOVA with eye, group and spatial frequency (SF) as factors revealed significant effects of group (F(1,64) = 11.46, *P* = 0.001), SF × group interaction (F(1.98, 126.76) = 7.08, *P* = 0.001), and SF × eye interaction (F(2.19, 140.31) = 5.70, *P* = 0.03), but no significant effects of eye (F(1, 64) = 0.01, *P* = 0.921), eye × group interaction (F(1,64) = 0.15, *P* = 0.703), and eye × group × SF interaction (F(2.19,140.31) = 0.79, *P* = 0.464). VKH patients had worse CSF than controls, especially at 3, 6, 12 and 18 cpd (Bonferroni corrected pairwise comparisons, all *P*s < 0.05).

There were significant effects of group on AULCSF (F(1,64) = 12.60, *P* < 0.001) and CSF acuity (F(1,64) = 10.31, *P* = 0.002) in the VKH group. The mean values of both metrics were markedly decreased in the VKH group than those of the controls (Bonferroni corrected pairwise comparisons, *P* < 0.05) (Fig. 2).

The average CSFs of the SGF, non-SGF and control groups are shown in Fig. 2. Repeated measures ANOVA revealed significant effects of group (F(2,63) = 7.79, *P* < 0.001), SF × group interaction (F(3.96,124.58) = 4.22, *P* = 0.003), and SF × eye interaction (F(2.17,136.53) = 5.67, *P* = 0.003), but no significant effects of eye (F(1,63) = 0.06, *P* = 0.815), eye × group interaction (F(2,63) = 0.08, *P* = 0.921), eye × group × SF interaction (F(4.33,136.53) = 0.76, *P* = 0.563). As shown in Fig. 2, patients with SGF had the worst CSF. Compared with the controls, the SGF group had the lower CSF, especially at 3, 6, 12 and 18 cpd (Bonferroni corrected post hoc tests, all *P*s < 0.05). No statistically significant difference was found between the non-SGF group and controls, nor between the SGF and non-SGF groups.

Considering the SGF, non-SGF and control groups, there were significant effects of the group on AULCSF (F(2,63) = 8.04, *P* < 0.001) and CSF acuity (F(2,63) = 7.62, *P* = 0.001). The mean values of both metrics in the SGF group were significantly lower than those of the controls (Bonferroni corrected post hoc tests, both *P*s < 0.001). The mean values of both metrics in the non-SGF group were lower than those of the controls, but the differences were not statistically significant (Bonferroni corrected post hoc tests, both *Ps* > 0.05). No statistically significant difference of either metric was found between the SGF and non-SGF groups (Bonferroni corrected post hoc tests, both *P*s > 0.05).

### Chorioretinal thickness in VKH patients

Chorioretinal thickness of the VKH patients and controls were analysed with repeated measures ANOVA with eye and group as factors. For the ORT, there was significant effect of group (F(1,64) = 23.13, *P* < 0.001). However, no significant effects of group in ONLT (F(1,64) = 0.29, *P* = 0.592) or CT (F(1,64) = 3.89, *P* = 0.053) were found. That is, VKH patients had significantly decreased ORT compared to the controls (Bonferroni corrected pairwise comparisons, *P* < 0.001) (Table 2).

**Table 2:** Chorioretinal thickness of the convalescent VKH patients and controls.

Parameters	Control	VKH	Non-SGF	SGF	*P*-value	*P*^a^-value	*P*^b^-value	*P*^c^-value
Outer nuclear layer thickness (μm)	106.31 ± 13.38	107.97 ± 13.84	107.89 ± 14.21	108.07 ± 13.59	0.592	1.000	1.000	1.000
Outer retinal thickness (μm)	112.59 ± 5.41	105.55 ± 7.49	107.06 ± 5.03	103.69 ± 9.47	<0.001	0.005	<0.001	0.237
Choroidal thickness (μm)	299.94 ± 73.17	334.69 ± 78.51	343.44 ± 80.89	323.88 ± 75.24	0.053	0.112	0.829	1.000

*P* = *P*-value between the control and VKH groups.

^*a*^*P* = *P*-value between the control and non-SGF groups.

^*b*^*P* = *P*-value between the control and SGF groups.

^*c*^*P* = *P*-value between the SGF and non-SGF groups.

To evaluate the influence of SGF, we compared the chorioretinal thickness of the SGF, non-SGF and control groups. Repeated measures ANOVA revealed a significant ORT difference between the groups (F(2,63) = 13.55, *P* < 0.001), but no significant ONLT (F(2,63) = 0.14, *P* = 0.866) and CT (F(2,63) = 2.29, *P* = 0.109) difference. Bonferroni corrected post hoc tests revealed that patients with (*P* < 0.001) or without (*P* = 0.005) SGF had thinner outer retina compared with the controls, but no significant difference between the two patient groups (*P* = 0.237) (Table 2).

### Relationship between CSF and chorioretinal thickness

Correlations between CSF metrics and chorioretinal thickness were evaluated using GEE with eye as a within-subject factor. No significant correlation was found between CSF metrics (CS at six spatial frequencies, AULCSF and CSF acuity) and chorioretinal thickness (ONLT, ORT and CT) in the VKH, SGF, and non-SGF groups (Table 3). When we considered the VKH patients and controls as a whole, we found significant correlation between CSF metrics (AULCSF and CSF Acuity) and ORT (β = 0.005, 95%CI = 0.001, 0.008, *P* = 0.005; β = 0.176, 95%CI = 0.065, 0.287, *P* = 0.002 respectively).

**Table 3:** Correlations between CSF metrics and chorioretinal thickness.

Parameters	Groups	Outer nuclear layer thickness (μm)			Outer retinal thickness (μm)			Choroid thickness (μm)		
		β	(95%CI)	*P*-value	β	(95%CI)	*P*-value	β	(95%CI)	*P*-value
AULCSF	VKH	<0.001	(−0.004, 0.003)	0.973	0.001	(−0.002, 0.005)	0.448	<0.001	(−0.0004, <0.001)	0.740
	SGF	−0.001	(−0.006, 0.005)	0.853	0.001	(−0.003, 0.006)	0.495	<0.001	(−0.0005, 0.001)	0.417
	Non−SGF	0.001	(−0.003, 0.004)	0.759	−0.004	(−0.009, 0.002)	0.215	−0.0002	(−0.001, <0.001)	0.476
CSF Acuity	VKH	0.041	(−0.048, 0.130)	0.370	0.103	(−0.022, 0.228)	0.106	0.004	(−0.010, 0.018)	0.558
	SGF	0.069	(−0.098, 0.236)	0.420	0.137	(−0.036, 0.310)	0.120	0.009	(−0.014, 0.032)	0.455
	Non-SGF	0.027	(−0.050, 0.104)	0.493	−0.159	(−0.339, 0.022)	0.084	−0.003	(−0.020, 0.014)	0.707

## Discussion

In this study, we measured CSF and chorioretinal structure in convalescent VKH patients as well as normal participants. We found reduced CSF and thinned outer retina in convalescent VKH patients. Eyes with SGF had more reduced CSF and chorioretinal thickness compared to those without SGF. Compared with controls, CSF was significantly impaired in eyes with SGF, especially at medium and high spatial frequencies. No significant correlation was found between CSF metrics and chorioretinal thickness.

The visual system contains many spatial frequency channels [44]. Whereas low and medium spatial frequency channels are important for object recognition in the real world (objects faces and road signs) [15, 45], high spatial frequency channels are critical for visual details discrimination [18, 45]. Previous research found that, for patients with uveitis, CS measured by the Pelli-Robson chart showed a negative correlation with the Vision Quality of Life questionnaire [46, 47]. The observed CSF deficits in medium and high spatial frequencies suggest VKH patients, especially those with SGF, may adapt to the basic visual demands, but have limitations in resolving the fine details of everyday objects.

We found significant CSF reduction only in eyes with SGF. The results suggest that patients with SGF were more likely to retain CSF dysfunction, despite good recovery of visual acuity. The presence of SGF may be linked to inflammatory recurrence [4, 10, 12]. The RPE cells provide nutrients to photoreceptors and participate in the visual retinoid cycle, which plays an important role in contrast detection [48]. SGF reflects depigmentation of the RPE and choroid, and the deficient protective barriers may affect the function of the foveal cone system [49, 50]. Okamoto et al. [49] found that photopigment regeneration was impaired, and its recovery took more time than focal macular electroretinogram parameters, colour vision and visual field in VKH patients with good visual acuity and intact macular morphology. Nakamura et al. thought the inflammation and serous retinal detachment caused the injury of outer segments of the photoreceptors [51]. Yang et al. [16] inferred that photoreceptor dysfunction may be a direct consequence of VKH disease or a secondary result of damage to the choroid and/or RPE. The impairments of visual function have also been observed in other depigmentary conditions. For example, typical retinitis pigmentosa patients may have potentially good acuity but poor contrast sensitivity [52]. Study have shown that, due to the absence of RPE melanin granules, albino neural retina optical absorption was at least 30% higher than that of normal retina [53]. Albinos are linked with poor visual function and photophobia with persistent photon damage [54]. We speculate that chorioretinal depigmentation may have led to reduced CSF in patients with SGF.

We found that the outer retina of patients with or without SGF was significantly thinner than that of the controls. Moreover, eyes with SGF had the thinnest outer retina, with disease progression. No significant ONLT difference was found among the groups. Although early retinal detachment had recovered in convalescent VKH patients, there may be other microstructural changes. Nakamura et al. evaluated the macular cone density in VKH patients using adaptive optics fundus camera, and they reported that the outer segment of the photoreceptor was damaged, but the final visual acuity was well maintained after 12 months therapy [51]. Zhu et al. reported the outer nuclear layer attenuation, interruption of myoid zone, ellipsoid zone, and outer segments of photoreceptors in convalescent VKH patients [55]. Our results showed that the changes predominantly occurred in ORT. No significant difference in CT was found among patients with or without SGF and healthy controls, consistent with previous studies [30, 56]. Jaisankar et al. showed the choroid remodelled and tended to normalize in VKH patients after systemic therapy [57]. However, Takahashi et al. found that CT was significantly reduced in patients with severe SGF [30]. One possible reason for this discrepancy is that different types of patients were recruited in the two studies. Takahashi et al. included patients with long durations of the disease (more than 3 years) and most patients with severe SGF also exhibited peripapillary atrophy. In this study, we only recruited patients with good visual acuity, whose choroidal impairment and fundus depigmentation were relatively mild.

Nakamura et al. indicated that the presence of subretinal fluid did not delay the functional recovery of photoreceptors in patients with VKH disease [51]. Abu et al. found no association between exudative retinal detachment and the final mean retinal sensitivity of 7.0 dB or better with logistic regression analysis [5]. In addition, the previous macular involvement in retinal detachment during the active phase of our participants was determined by the optical coherence tomography images or the medical record. There were missing data for 10 eyes in the SGF group and for 6 eyes in the non-SGF group. Macula-involving retinal detachment was found in 17 eyes in the SGF group and 32 eyes in the non-SGF group. No macula-involving retinal detachment was found in 7 eyes in the SGF group and 4 eyes in the non-SGF group. The data were analysed by Chi-square test (*X*^2^ = 2.05, *P* = 0.15). There was no significant difference between the two groups. Thus, it is less likely that the previous macular involvement in retinal detachment during the active phase contributed to our results.

Contrast detection relies on intact chorioretinal structure [24, 26, 58]. Correlations between CSF metrics and macular structure have been reported in other eye diseases [19, 24, 26, 59]. In a cross-sectional study, late-stage VKH patients with intact cone outer segment tips line had better retinal sensitivity measured with MP-1 microperimeter [28]. However, our study did not find any significant correlation between CSF metrics (AULCSF and CSF acuity) and structural thickness (ORT, ONLT and CT) in eyes with or without SGF. The relationship between CSF metric and chorioretinal thickness might be more complicated than a simple linear relationship [60]. Although more detailed structure stratification was not performed in this study, we did notice ellipsoid zone loss, discontinuous RPE signal or RPE folds in several patients. Recently, studies showed fundus structure damages in VKH disease at the cellular and ultrastructural levels, or in terms of microvascular circulation [55, 61–63]. More detailed multidimensional approaches might be necessary to further evaluate the relationships between CSF and retinal structure in patients with VKH disease.

## Conclusions

To conclude, despite good recovery of visual acuity, reduced CSF and outer retina thickness were found in convalescent VKH patients. CSF may be an important and sensitive metric to evaluate functional vision in VKH disease.

## Summary

### What was known before

- Clinically, patients with recovered visual acuity still complain about poor visual quality.

### What this study adds

- Despite good recovery of visual acuity, reduced contrast sensitivity function and outer retina thickness were found in convalescent Vogt-Koyanagi-Harada patients.

## Data Availability

The data used during this study are available from the corresponding author on reasonable request.
